# Multifocal skull-compensated transcranial focused ultrasound system for neuromodulation applications based on acoustic holography

**DOI:** 10.1038/s41378-023-00513-3

**Published:** 2023-04-10

**Authors:** Geon Kook, Yehhyun Jo, Chaerin Oh, Xiaojia Liang, Jaewon Kim, Sang-Mok Lee, Subeen Kim, Jung-Woo Choi, Hyunjoo Jenny Lee

**Affiliations:** 1 School of Electrical Engineering, Korea Advanced Institute of Science and Technology, 291 Daehak-ro, Yuseong-gu, Daejeon, 34141 South Korea; 2 KAIST Institute for NanoCentury (KINC), 291 Daehak-ro, Yuseong-gu, Daejeon, 34141 Republic of Korea

**Keywords:** Electrical and electronic engineering, NEMS

## Abstract

Transcranial focused ultrasound stimulation is a promising therapeutic modality for human brain disorders because of its noninvasiveness, long penetration depth, and versatile spatial control capability through beamforming and beam steering. However, the skull presents a major hurdle for successful applications of ultrasound stimulation. Specifically, skull-induced focal aberration limits the capability for accurate and versatile targeting of brain subregions. In addition, there lacks a fully functional preclinical neuromodulation system suitable to conduct behavioral studies. Here, we report a miniature ultrasound system for neuromodulation applications that is capable of highly accurate multiregion targeting based on acoustic holography. Our work includes the design and implementation of an acoustic lens for targeting brain regions with compensation for skull aberration through time-reversal recording and a phase conjugation mirror. Moreover, we utilize MEMS and 3D-printing technology to implement a 0.75-g lightweight neuromodulation system and present in vivo characterization of the packaged system in freely moving mice. This preclinical system is capable of accurately targeting the desired individual or multitude of brain regions, which will enable versatile and explorative behavior studies using ultrasound neuromodulation to facilitate widespread clinical adoption.

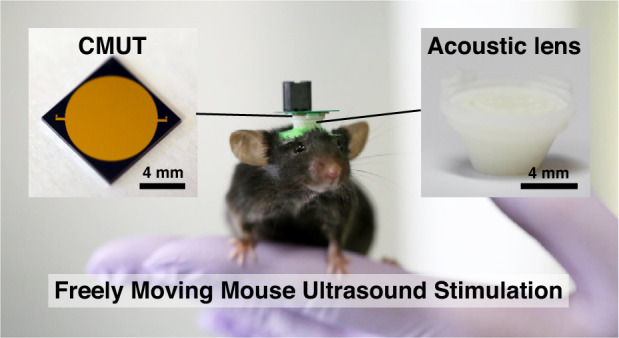

## Introduction

Among several biomedical applications of ultrasound, transcranial focused ultrasound stimulation (TFUS) has emerged as a new application with great potential in neuroscience^1–3^. Because of its unique advantages over conventional stimulation technologies (e.g., optogenetics, transcranial direct current stimulation (TDCS), and transcranial magnetic stimulation (TMS)) including noninvasiveness, long penetration depth, and versatile spatial control with high spatial resolution, TFUS is applicable to multiple domains of fundamental, clinical, and translational research^4–9^. Focused ultrasound noninvasively delivers acoustic energy to specific regions of the brain with sufficient energy, and locally modulates neural activities via mechanosensitive ion channels on neurons^10–13^. In addition to direct ultrasound neuromodulation, ultrasound can also be used for noninvasive energy delivery and brain stimulation. Through combination with energy-converting nanoparticles, such as piezoelectric nanoparticles^14,15^ and mechanoluminescent nanoparticles^16^, transcranial ultrasound can noninvasively achieve electrical and optical stimulation with high spatial resolution.

While TFUS offers high clinical potential, a major bottleneck precluding its application in translational research is the unknown effects of neuromodulation therapy. A large set of explorative studies is needed to address this issue, but the lack of a preclinical ultrasound system suitable for behavioral studies of mice with versatile focusing capabilities hinders the realization of such studies. Recently, preclinical neuromodulation systems based on miniature transducers such as capacitive micromachined ultrasound transducers (CMUT)^17,18^ and piezoelectric ultrasound transducers (PUT)^19,20^ have been proposed to enable behavioral studies with ultrasound neuromodulation. However, these early works lacked compensation for focal aberration.

Focal aberration is an important challenge in focusing ultrasound accurately on a targeted brain through a skull. While focal aberration by a mouse skull is a minor issue for ultrasound frequencies under 1 MHz^21,22^, it is severe and must be accounted for during stimulation of target subregions requiring excitation by frequencies higher than 5 MHz^23^. Focal aberration can be compensated to generate the desired stimulation by calculating and manipulating the wavefront of the ultrasound beam using acoustic holography^24–26^. Conventionally, wavefront manipulation has been implemented using phased array transducers (PATs), where individual transducers are driven by excitation signals of different phases^25^. However, it has been difficult to utilize PATs for brain stimulation in mice because of the large size of the actuation device and interface. A phased array with a large number of transducers often requires complex wiring to interface and thus is not optimal for preclinical studies that involve behavioral studies in mice^27,28^. Therefore, the single-curved transducer, which focuses ultrasound in free space using a single-channel interface, has been primarily used for brain stimulation in small animals without compensation of focal aberration^13,19,22,29^.

An alternative method of focusing ultrasound is to utilize an acoustic lens with a single-channel transducer. This acoustic lens is ultralight, simple in structure, and equipped with a single transducer powered by a single pair of electrical connections. Therefore, it is highly suitable for neuromodulation in freely moving small animals, especially mice. Recently, using 3D-printing technology, acoustic lenses with various 3D microscale structures have been fabricated and utilized for phase modulation of acoustic waves in large-sized systems^30,31^. The acoustic lens was also applied to the neuromodulation of small-sized animals and demonstrated its multitargeting ability in the brain^32^. However, its application was limited to realizing acute stimulation under anesthesia within stereotaxic fixation, due to the use of a bulky transducer. Thus, there remains an unmet need for a preclinical system with versatile spatial control. The previously reported works either lack focusing^17^, skull-aberration compensation^19,20^, or in vivo characterization of the freely behaving package;^13,32–34^ they also exhibited poor spatial resolution^17,18^ and were bulky in size^27,28,31,32^, thus being unsuitable for chronic behavioral studies of mice with abundant disease models.

**Fig. 1:**
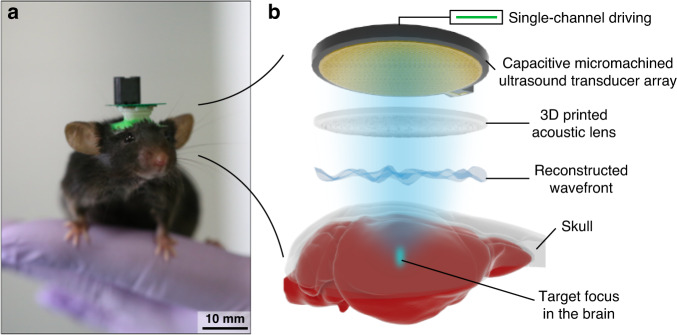
Overview of the ultralight miniature focused ultrasound brain stimulation system with versatile focusing capability. **a** Photograph of the complete transcranial system mounted on a freely behaving mouse. **b** Conceptual schematic diagram of the overall system and its components with the visualization of the sound beam focused through the MEMS ultrasound transducer and 3D-printed acoustic lens

Here, we demonstrate a fully functional, preclinical ultrasound brain neuromodulation system for behavioral studies of mice with versatile spatial control through beamforming and beam steering. We utilize MEMS and 3D-printing technology to implement a 0.75-g lightweight neuromodulation system suitable for behavior studies of rodents. An acoustic lens acting as a time-reversal mirror^31^ was designed through acoustic simulation with modeled skulls and fabricated by stereolithographic 3D-printing. A CMUT acting as a miniaturized planar acoustic source was designed and fabricated by wafer-to-wafer bonding and silicon micromachining processes. In CMUTs, ultrasound is generated by the vibration of a micromachined membrane structure fabricated on a wafer^35^. Unlike conventional PUTs, CMUTs can operate in a standalone chip-sized form factor without the need for a housing, backing, and matching layer^19^. For CMUTs fabricated on a silicon substrate with a thickness of 725 µm, CMUTs weigh only ~0.17 g per aperture area of 1 cm^2^. Thus, even small animals such as mice can carry CMUTs without restrictions, which enables a large set of behavioral studies to investigate the effects of ultrasound stimulation in freely behaving animals. The total weight of the manufactured package, including the transducer and acoustic lens, was only 0.75 g, which imparted no significant change to the mouse behavior (Fig. 1). Using our system, users can target subregions of the brain with high accuracy and achieve multitargeting in explorative behavior studies using ultrasound neuromodulation.

## Experimental methods and calculations

### Time-domain ultrasound simulation of the time-reversal recording involving skulls

The differential equations for an acoustic wave in inhomogeneous absorbing media are as follows:

1 $$\frac{{\partial \rho }}{{\partial {{{\boldsymbol{t}}}}}} \,=\, - \rho _0\nabla \cdot {{{\boldsymbol{u}}}} \,-\, {{{\boldsymbol{u}}}} \cdot \nabla \rho _0$$

2 $$\frac{{\partial {{{\boldsymbol{u}}}}}}{{\partial {{{\boldsymbol{t}}}}}} \,=\, - \frac{1}{{\rho _0}}\nabla {{{\boldsymbol{p}}}}$$

3 $$p \,=\, c_0^2\left( {\rho \,+\, {{{\boldsymbol{d}}}} \cdot \nabla \rho _0 \,-\, L\rho } \right)$$

where *p* is the sound pressure, *ρ*_0_ is the static density of the medium, *ρ* is the acoustic density, ***u*** is the particle velocity, ***d*** is the particle displacement, *c*_0_ is the speed of sound in the media, and *L* is a linear operator defined as:

4 $$L \,=\, - 2\alpha _0c_0^{\gamma - 1}\frac{\partial }{{\partial t}}\left( { - \nabla ^2} \right)^{\frac{\gamma }{2} - 1} \,-\, 2\alpha _0c_0^\gamma \tan \left( {\frac{{\pi \gamma }}{2}} \right)\left( { - \nabla ^2} \right)^{\frac{{\gamma - 1}}{2}}$$

where *α*_0_ is the absorption coefficient of the medium and *γ* is the exponent of the frequency power law of absorption^36–39^. To solve the acoustic differential equations in the aberrating media involving skulls, a simulation tool was developed using k-Wave, which is an open-source acoustic toolbox for MATLAB (Mathworks, USA) that numerically solves the equation based on the k-space pseudospectral approach. The simulation tool was used to obtain accurate numerical results with a smaller number of temporal and spatial steps, for lower computational costs^40–43^.

First, a three-dimensional space was built with spatial grids smaller than half of the wavelength of the ultrasound. Using 3D models of skulls from X-ray computerized tomography (CT) imaging of real skulls (Mouse Imaging Center, Canada), skull models were imported into grid points of the 3D model mesh. Acoustic properties, such as medium density, speed of sound, and acoustic attenuation coefficient, were assigned to represent bone inside the mesh and water (or tissue) elsewhere. Typical acoustic characteristics of the bone were assigned based on values reported in reference^44^. With the acoustic properties assigned, sound propagation was simulated from a sound source at a target position. To observe a sinusoidal steady state, 20 (or more) sinusoidal pulses of acoustic pressure were generated at the source. The size of the temporal step was given as a Courant–Friedrichs–Lewy (CFL) number of 0.3, which describes the ratio of the distance a wave can travel in one time step over the grid spacing. The time-domain signal of acoustic pressure was recorded at sensors that were placed where the transducer was positioned. After the system reached a sinusoidal steady state, the phase of the acoustic pressure was obtained.

### Design and fabrication of the acoustic lens

Using the principle of a phase conjugation mirror, the acoustic lens was designed to reconstruct the conjugation of the recorded phase from a simulated time-reversal recording for focusing the target. By interfacing two media, medium A and medium B, the phase difference at a coordinate arises from a reference point where the thickness of medium A is *T*_0_ as follows:

5 $$\Delta \phi \left( {x,y} \right) \,=\, \left( {k_B - k_A} \right)\left( {T_0 \,-\, T\left( {x,y} \right)} \right)$$

where *T*(*x,y*) is the thickness of medium A at the given coordinate, and *k*_*A*_*, k*_*B*_ are the wavenumbers in medium A and B, respectively^30^. After converting the phase profile to a 3D model of the acoustic lens, a stereolithography (SLA)-type 3D printer (Form3, Formlabs, USA) was used to fabricate the acoustic lens using a UV-curable resin (White, Formlabs, USA). The spot size of the laser was 85 µm, and the xy-resolution was 25 µm. The three-dimensional structure of the fabricated acoustic lens was analyzed using an X-ray microscope (Xradia 520 Versa, Zeiss, Germany). The focal size of the X-ray was 10 µm for the void analysis and 50 µm for the three-dimensional profile analysis. Porosity was evaluated as voxels with the lower 10% of the X-ray transmittance in the volume. Three-dimensional reconstruction of the lens was qualitatively performed by manual transmittance thresholding. A cross section of the fabricated lens was analyzed using a scanning electron microscope (SEM) (S-4800, Hitachi, Japan).

### Measurement of the ultrasonic beam profile

The ultrasonic beam profile was measured by scanning a needle hydrophone (NHO500, Precision Acoustics, United Kingdom) over the volume of interest. The position of the hydrophone was controlled by a 3D motor system for precise scanning. The measured voltage from the hydrophone was converted to acoustic pressure using calibrated sensitivity values of the hydrophone at the given frequency. For assessment of the focal correction of the acoustic lens, the coordinate of the scanning volume was referenced to the target by optically aligning the needle hydrophone to slits on the skull phantom, which was designed to enable this alignment.

### Fabrication of the capacitive micromachined ultrasound transducers (CMUT)

The CMUT was fabricated using a wafer bonding process^45^. First, a heavily boron-doped 100-mm-diameter Si wafer (<0.005 Ω ∙ cm) was thermally oxidized under wet conditions to grow 100 nm of SiO_2_ as an insulation layer. Next, a separate 100-mm-diameter silicon-on-insulator (SOI) wafer with a 1.2-µm-thick active layer was thermally oxidized under wet conditions to grow 150 nm of SiO_2_. The SiO_2_ layer in the SOI wafer was etched in a 6:1 buffered-oxide-etch (BOE) solution to form cavities in the CMUT. The surfaces of the two wafers were activated by immersion in a 5:1:1 (v/v/v) mixture of H_2_O, 29% NH_4_OH (aq), and 31% H_2_O_2_ (aq) for 15 m at 70 °C and then in a 3:1 (v/v) mixture of 98% H_2_SO_4_ (aq) and 31% H_2_O_2_ (aq) for 15 m at 70 °C. The two wafers were bonded through fusion bonding at the SiO_2_–SiO_2_ interface in a vacuum.

After bonding, the bonded wafer was annealed for 4 h at 1050 °C. The handling Si layer on the SOI wafer was ground to leave approximately 30 µm of Si, and the remaining Si was completely removed in a 5% (CH_3_)_4_N(OH) (tetramethylammonium hydroxide, TMAH) aqueous solution at 80 °C. Then, the exposed buried oxide (BOX) layer on the SOI wafer was removed in a 49% (w/w) aqueous solution of HF. The device layer of the SOI wafer was formed as a single-crystalline Si membrane of the CMUT. The membrane was etched for the isolation of each device using reactive ion etching (RIE). By etching the SiO_2_ layer, the highly doped Si substrate from the first wafer was exposed to form the bottom electrode. Finally, electron-beam evaporation and etching of a 5/100-nm Cr/Au layer served as top and bottom electrodes and pads for the operation of the CMUT (Fig. S8).

### Measurement of the transducer bandwidth

The CMUT was immersed in oil for ultrasound generation to mimic the water environment with electrical insulation. With a DC bias of 42 V, a 100-ns-wide rectangular pulse (with a frequency-domain plateau greater than −3 dB in the 0–5 MHz range) with 10 V amplitude was applied to drive the CMUT to observe its frequency response under 5 MHz. The impulse response of the CMUT was measured using a needle hydrophone (NHO500, Precision Acoustics, United Kingdom) with a uniform sensitivity in the frequency range of 1–5 MHz. The signal from the hydrophone was processed through a preamplifier and a DC coupler, and the voltage values were measured using an oscilloscope. The response signal was Fourier transformed, and the frequency-domain response characteristics of the device were analyzed.

### Packaging of the CMUT and acoustic lens

PDMS (Sylgard 184, Dow, USA) and curing agent were mixed in a 10:1 (w/w) ratio and degassed in a vacuum desiccator with the CMUT and the lens immersed in the mixture. The CMUT and the lens were carefully combined in the mixture to avoid air bubbles at the interface. To align the lens to the aperture of the CMUT, a guiding structure was designed on the lens. The CMUT-lens package was cured at 70 °C for 6 h in a vacuum oven. The electrodes of the CMUT were electrically connected through wire bonding to the pads on the 0.2-mm-thick PCB, and flexible copper wires were soldered to the PCB to drive the CMUT. Epoxy resin was used to insulate the exposed solders and wire-bonded pads.

### Animal care

All animal experiments conducted in this study were in accordance with protocols outlined and approved by the Institutional Animal Care and Use Committee at the Korea Advanced Institute of Science and Technology (KA2021-039). Animals were housed in individual cages with food and water available *ad libitum* under a 12-h light/dark cycle, with lights turned on at 07:00. A total of 9 male C57BL/6J mice (6–8 weeks old) were used in the experiments.

### In vivo device packaging

Mice were anesthetized with isoflurane (4% for induction and 1.5% for maintenance) and head-fixed in a stereotaxic frame (RWD Life Science Co., Ltd., China). Ophthalmic ointment and lidocaine were applied for the prevention of eye drying and pain reduction during the surgery. The fur on the scalp was gently removed, and the scalp was carefully excised to expose the skull. Medical-grade saline was used to clean the surface of the skull. By referencing the tip of the collimator to the bregma, the packaged CMUT device was positioned above the target coordinates. The device was fixed to the skull using Loctite adhesive. The exposed skull was protected by casting biocompatible silicone elastomer (Kwik-Cast, World Precision Instruments, USA).

### Freely moving behavior experiment

Mice were given 7–10 days of recovery postsurgery with food and water provided ad libitum. All animals were carefully handled by two experimenters for 5 min per day over 3 days during the recovery period prior to conducting any behavior tests. Handling habituation was determined by each mouse displaying grooming behavior in the hands or after a 5-min exploration across the arms. We conducted freely moving behavioral tests in a custom-made square open field (20 × 30 × 30 cm) to assess the impact of our chronically attached device on locomotion (NANUM Design, Daejeon, Korea). Three experimental groups were used to determine the effects of surgery and device placement on the speed and total distance traveled by the mice: (1) Normal group (without device and surgery), (2) Sham group (without device but with sham surgery), and (3) Device group (with device and surgery). For the Device group, we untethered the device to observe the effects of the device weight independently. For the Sham group, the same surgical procedure was followed, replacing the device attachment step with silicone elastomer. Each experiment consisted of a brief handling period immediately followed by a 3-min freely moving session in the open field. The experiment was repeated 2–3 times per mouse with 70% ethanol used to clean the chamber between each term.

### In vivo behavior analysis

To evaluate the freely moving behavior of the mice, DeepLabCut (DLC) was used, which is a widely used open-source software package for animal pose analysis based on machine learning algorithms^46^. A Linux system running Ubuntu 16.04 and equipped with an AMD Ryzen 7 1700, 32 GB of RAM, and NVIDIA TITAN Xp was used for the computations. A representative video of a control mouse was recorded for training, and the body of the mouse was manually labeled for 19 frames. Using the ResNet-50 convolutional neural network (CNN), the DLC network was trained for 300,000 iterations. The trained network was used to label the movement of the mouse for each of the 180-s videos. The movement trajectory of the mouse was processed to evaluate the movement behavior of the mouse. The average speed was calculated by dividing the aggregate square root distances of each trajectory point by the total time. Cumulative travel distance was evaluated by integration of the displacement of the mouse between each frame. At a single frame, the occupancy rate of the mouse in the pixels (x, y) was evaluated as a Gaussian weighing with σ of 1 cm from the trajectory:

$$A \,=\, \exp \left( { - \frac{{x^2 \,+\, y^2}}{{2\sigma ^2}}} \right)$$

The total occupancy rate of the mouse was evaluated by integrating the occupancy rate over the entire frame.

### Mouse skull extraction

The cranium was harvested from cardiac-perfused mice following the same protocol as previously published^13^. The brain was removed, and the ventral half of the skull was excised away to open the skull cavity for hydrophone insertion. The dorsal half of the skull was carefully washed in medical-grade saline and positioned relative to bregma for precise beam profile measurements.

### Statistical analysis

Statistical analysis of behavioral data was conducted using one-way analysis of variance (ANOVA) with Tukey’s post hoc test. For one-way ANOVA, the experimental group was used as the factor. All data were analyzed using Microsoft Excel (Microsoft Co., Redmond, WA, USA), OriginPro 2019 (OriginLab Co., Northampton, MA, USA), and MATLAB R2022a (The MathWorks Inc., Natick, MA, USA). All data are presented as the means ± standard errors of means (s.e.m.), and a *p*-value of <0.05 is considered significant.

## Results and discussion

### Fabrication and characterization of the skull-compensated acoustic lens

**Fig. 2:**
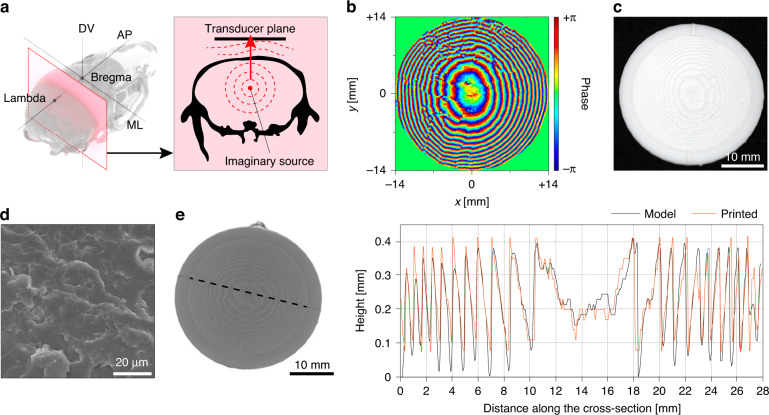
Design and fabrication of the skull-compensated acoustic lens. **a** Conceptual diagram of the simulation based on the time-reversal mirror across a mouse skull. **b** Example of the recorded phase from the simulated time-reversed signal. **c** Optical image of the 3D-printed acoustic lens designed to reconstruct the phase in (**b**). **d** Scanning electron microscopy (SEM) image of the cross section of the 3D-printed acoustic lens. **e** Surface profile along the black dotted line of the 3D-printed acoustic lens from X-ray microscopy

To calculate the acoustic wavefront with an accurate focus in the brain area behind the skull, we adopted the principle of a time-reversal mirror (Fig. 2a). A numerical approach of the k-space pseudospectral method was utilized for the time-domain simulation of the acoustic wave with the consideration of absorptive propagation through the skull^36–39^. The geometry of the skull was imported from the 3D mesh files from X-ray computerized tomography (CT) data (Mouse Imaging Center, Canada) and was assigned to spatial grids. An acoustic point source was positioned at the target position inside the skull cavity to drive sinusoidal acoustic pressure, and the time-domain signal of the acoustic pressure was recorded on the source plane configured at the position of the actual ultrasound transducer. Phase information obtained from the recorded time-domain acoustic pressure signal was conjugated to obtain an excitation phase map that would focus ultrasound at the target position (Fig. 2b).

To generate an ultrasound field based on the calculated excitation phase map, we developed a miniaturized 3D-printed acoustic lens that interfaces with a single planar ultrasound transducer. At short traveling distances, sound transmission through the thin lens was modeled as a one-dimensional propagation with only a phase delay proportional to the thickness of the lens^30^. Using 3D printable resins for the lens (density, ρ = 1170 kg m^-3^, acoustic impedance, Z = 3.03 MRayl), the thickness of the acoustic lens decreases to 1 mm to achieve the maximum phase delay of 2π for ultrasound frequencies over 1 MHz. Since microscale fabrication with high spatial resolution under 100 µm is possible using conventional 3D printers, we successfully implemented a miniature 3D-printed acoustic lens and achieved fine phase modulation (with pixel size down to ~0.01 mm^2^ and phase modulation unit down to ~2π/10) with only a single-channel transducer to replace conventional PATs for transcranial focusing^30–32^. From the previously obtained phase map, a 3D model of the acoustic lens was generated in STL format and fabricated using an SLA (stereolithography)-type 3D printer (Form3, Formlabs, USA) (Fig. 2c). Because the SLA-type 3D printer results in minimal internal print voids^47,48^, no evident voids were observed in the fabricated acoustic lens under X-ray microscopy (Xradia 520 Versa, Zeiss, Germany) (Fig. S1) and scanning electron microscopy (SEM) (S-4800, Hitachi, Japan) (Fig. 2d). The surface profile of the fabricated lens was also analyzed by X-ray microscopy and compared to the ideal profile of the input model of the 3D printer (Fig. 2e). When the same acoustic lenses from different printing trials were analyzed to evaluate the repeatability of the fabrication, no evident deviation was noticed (Fig. S2). Although we carefully chose the thresholding values for X-ray transmittance when obtaining the model, it is noteworthy that these profile data are qualitative.

### Acoustic characterization of the skull-compensated acoustic lens

For evaluation of the developed acoustic lens, a skull phantom that mimics a real animal skull was manufactured using the 3D printer. The skull phantom was in the shape of an anatomical human skull with a halved size and was estimated to aberrate the ultrasound more than a real mouse skull in the simulation (Fig. S3). A 5-MHz commercial planar piezoelectric ultrasound transducer (PUT) with an aperture diameter of 25.4 mm was used for the evaluation. The transducer was then interfaced with the 3D-printed acoustic lens through an intermediate layer composed of silicone elastomer (Sylgard 184, Dow, USA) (Fig. S4). The acoustic lens used for this evaluation was designed to modulate the wavefront through the interface of the silicone elastomer and the resin to focus the ultrasound at a target inside the skull phantom. The skull phantom and lens-transducer package were immersed in a water tank and aligned using a 3D-printed holder (Fig. S5). The transducer was driven by single-channel sinusoidal pulses of voltage to generate ultrasound. To evaluate the effects of skull compensation, a Fresnel acoustic lens that focuses ultrasound without the correction of the skull phantom was also fabricated as a reference.

**Fig. 3:**
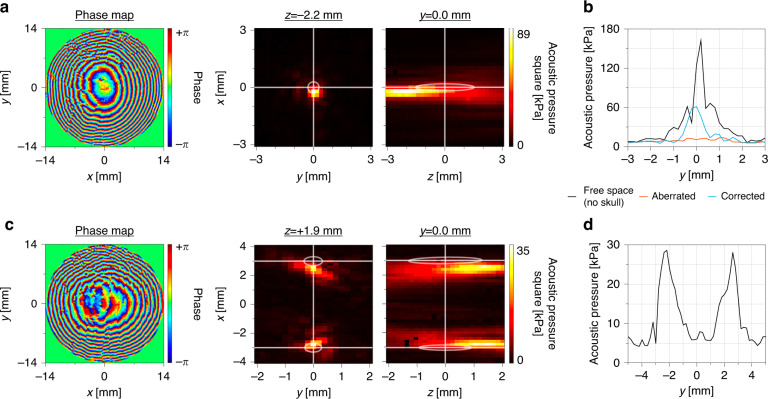
Characterization of the skull-compensated acoustic field from the fabricated acoustic lens. **a** Phase design of the skull phantom-compensated acoustic lens and the corrected acoustic pressure square profile from the fabricated lens. **b** Pressure square profile along the y-axis of the corrected beam, the aberrated beam, and the free-space focus as a reference. **c** Phase design of the skull phantom-compensated acoustic lens for two focal positions and the acoustic pressure square profile from the fabricated lens. **d** Pressure square profile along the y-axis of the beam in (**c**)

The focal profile obtained from the acoustic lens designed and fabricated using the time-reversal recording showed a clear ultrasonic focus through the skull phantom, while that from the Fresnel lens was highly aberrated (Figs. 3a, S6). Spatial deviations in the focal position were within the full-width at half maximum (FWHM) of the pressure square profile (Fig. 3b). The deviation was attributed to the alignment accuracy of the measurement system as well as the variation from the lens fabrication. Nonetheless, the developed acoustic lens was accurate enough for targeted brain stimulation even at a high frequency of 5 MHz, where skull correction is critical for small animals^23^. In addition, we successfully demonstrated the multifocusing ability of our lens system. By configuring two different point sources during the simulated time-reversal recording, it was possible to implement an acoustic lens that generates two foci of acoustic pressure, again using the spatial deviations within the FWHM (Fig. 3c, d). This multifocus acoustic lens is capable of targeting multiple regions in the brain using only a single-channel planar transducer and without the need for bulky PAT systems. By using our multifocal stimulation technique, TFUS could replace current invasive multisite electrical stimulations. For example, bilateral deep brain stimulation for Parkinson’s disease treatment could be achieved through noninvasive treatment using TFUS^49^. Finally, the steering ability of the acoustic lens^50^ was investigated to further verify the characteristics of the aberration-correcting acoustic lens (Fig. S7). We successfully focused the ultrasound at different positions inside the skull phantom, separated by 1 mm from the target simply by steering the lens.

### Fabrication of the MEMS ultrasound transducer

Next, we developed a capacitive micromachined ultrasound transducer (CMUT) as a planar ultrasonic transducer for our system. A CMUT is one type of acoustic transducer that consists of a micromachined membrane on a vacuum cavity^35^. By applying harmonic voltage perturbation with a DC bias on the membrane across the cavity, the membrane vibrates and pushes the medium to generate ultrasound. A nearly uniform planar wave can be produced from an array of micron-size CMUT cells when driven by identical signals. Because CMUT operates as a standalone chip without any additional structures such as backing and matching layers^51^, it is an excellent candidate for a miniaturized system that interfaces with the acoustic lens. Moreover, CMUTs can accommodate a wide range of ultrasound frequencies, and the resonance frequency of the vibrating structure can be readily adjusted by changing the membrane thickness and cavity radius.

**Fig. 4:**
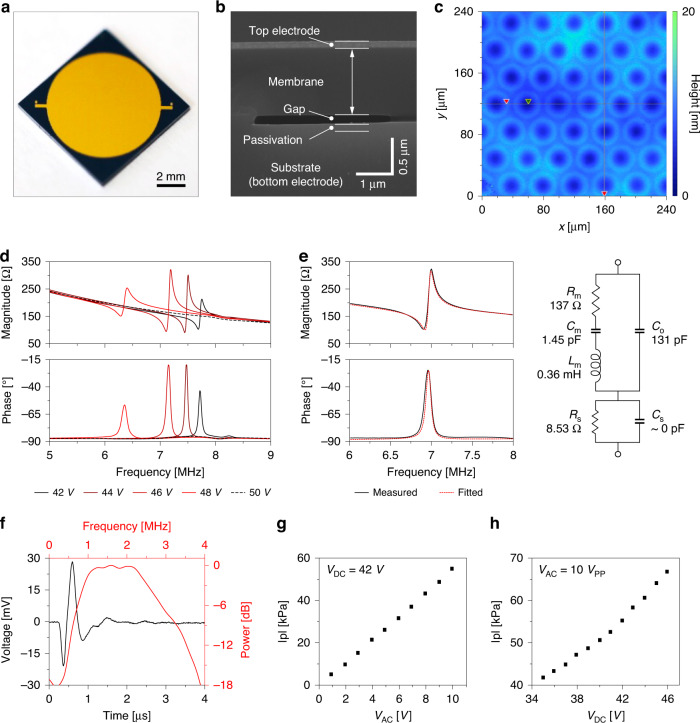
Characterization of the fabricated capacitive micromachined ultrasound transducer (CMUT). **a** Photograph of the fabricated capacitive micromachined ultrasound transducer (CMUT) with an aperture diameter of 8 mm. **b** Scanning electron microscope (SEM) image of the cross section of the fabricated CMUT. **c** 3D optical profile of the surface of the CMUT with static deflection of the membranes. **d** Electrical input impedance of the fabricated CMUT at different DC bias voltages in air. **e** 6-component modeling of the CMUT and the fitted values for each component (DC bias of 47 V). **f** Impulse response of the fabricated CMUT to a 10-V 100-ns-wide rectangular pulse measured by a needle hydrophone (DC bias of 42 V). **g** Output pressure of the CMUT measured in oil at a fixed point when driven with different peak-to-peak AC amplitudes with a DC bias of 42 V and **h** when driven with different DC bias voltages with a peak-to-peak AC amplitude of 10 V_pp_

Based on the analytical mass-spring model of the CMUT^52^, the transducer was designed with a silicon membrane of 1 µm thickness, a cell radius of 18 µm, and a gap height of 200 nm, yielding a theoretical resonance frequency of 8 MHz in air. The design was confirmed using a finite element model (FEM) simulation (COMSOL Multiphysics, COMSOL Inc., Sweden), and the resonance frequency was estimated to be 2 MHz in water. We fabricated the CMUT using the direct wafer bonding process with a high yield of 80% on a 100-mm silicon wafer (Figs. 4a, S8). The fabricated CMUT consisted of 35,683 individual cells placed on a circular aperture of 8 mm in diameter. All cells were electrically shorted to form a single device. The CMUT device was electrically interfaced through the top and bottom electrodes comprised of a gold film and highly boron-doped silicon, respectively. The cross-sectional scanning electron microscope (SEM) image confirmed that the fabricated CMUT consisted of a Si membrane of 0.97 μm thickness, a vacuum gap of 150 nm height, and a SiO_2_ insulation layer of 120 nm thickness (Fig. 4b). The static deflection of the silicon membrane was ~6 nm toward the vacuum cavity when measured by a 3D optical profiler (ContourGT, Bruker, MA, USA) (Fig. 4c).

### Electrical and acoustic characterization of the MEMS ultrasound transducer

To evaluate the resonance characteristics of the fabricated CMUT, the input impedance of the CMUT with different DC bias voltages of 42, 44, 46, 48, and 50 V was measured in air (Fig. 4d). The characteristic impedance peaks of the CMUT showed that the resonance frequency ranged between 7 and 8 MHz in air and decreased as the DC bias voltage increased due to the spring-softening effect^53^. The abrupt disappearance of the resonance peak over a DC bias over 50 V indicated the pull-in phenomenon of the silicon membrane in the CMUT. The measured electrical impedance of the CMUT in air was fitted to the 6-element equivalent circuit model of the CMUT, which includes mechanical components (R_m_, L_m_, and C_m_), a static capacitor (C_0_), and series parasitic components (R_s_, C_s_)^54^. The values of C_0_, R_m_, L_m_, C_m_, R_s_, and C_s_ were 131 pF, 137 Ω, 0.36 mH, 1.45 pF, 8.53 Ω, and 10^-13 ^pF (~0 pF), respectively (Fig. 4e).

For evaluation of the fabricated CMUT as an ultrasound transducer, the CMUT was actuated in oil and the acoustic pressure field was measured by the needle hydrophone. Using fast Fourier transform (FFT) analysis of the response signal for a 100-ns-wide rectangular pulse of 10 V amplitude with 42 V of DC bias, the center frequency and bandwidth were measured to be 2 MHz and 1.5 MHz, respectively (Fig. 4f). Additionally, the output acoustic pressure proportionally increased as the DC bias and AC voltage increased (Fig. 4g, h). Finally, the spatial profile of the acoustic pressure amplitude was measured with a driving peak-to-peak AC voltage of 10 V and a DC bias of 37 V at the center frequency of 2 MHz. We compared this spatial profile to the simulated profile of an ideal planar acoustic transducer of the same aperture diameter and confirmed that the fabricated CMUT acted as a planar acoustic source (Fig. S9). The maximum pressure amplitude was 87.7 kPa, which was larger than the minimum guideline for low-intensity ultrasound brain stimulation^55^. Considering that this value of acoustic pressure was obtained without focusing, the acoustic pressure was sufficiently large to be used for neuromodulation.

### Integration of the acoustic lens and the ultrasound transducer

**Fig. 5:**
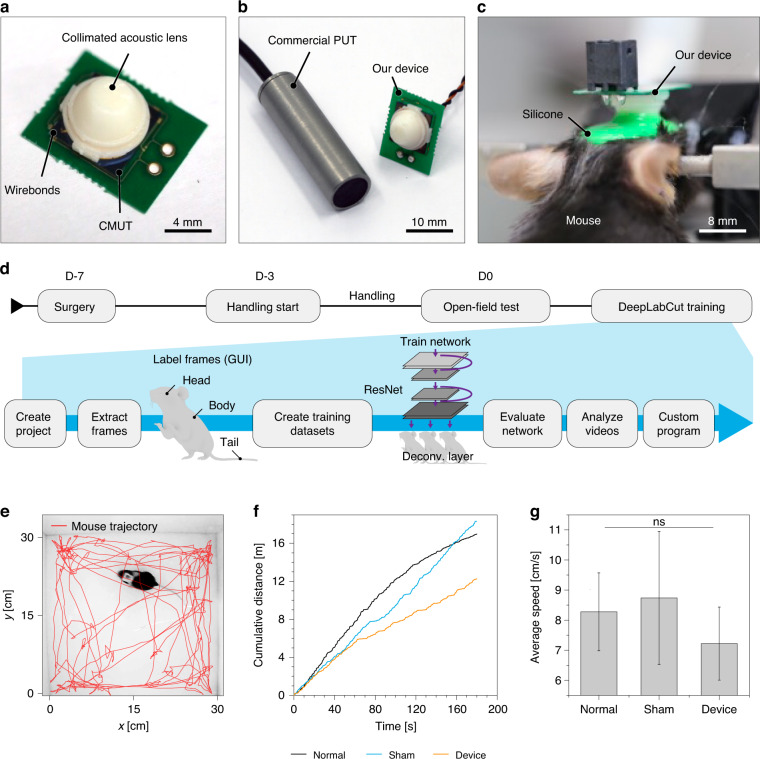
In vivo characterization of the integrated package of the acoustic lens and the capacitive micromachined ultrasound transducer (CMUT). **a** Photograph of the package composed of the capacitive micromachined ultrasound transducer (CMUT) and the acoustic lens. **b** Size comparison between the developed package and a commercial piezoelectric ultrasound transducer (PUT). **c** Photograph of the package of the developed device implanted on a mouse head. **d** Experimental timeline for observation of the freely moving behavior of the mouse with the inset showing the workflow schematics of DeepLabCut software to track movement. **e** Tracking of the position of a mouse in an open field using DeepLabCut. **f** Cumulative distance traveled by mice from three experimental groups. **g** Average speed of mice from three experimental groups. NS. indicates a nonsignificant *p*-value analyzed using one-way ANOVA with Tukey’s post hoc test (*p*-value > 0.05)

The fabricated CMUT was integrated with the acoustic lens to evaluate the in vivo characterization of the package. First, a collimator was designed as a conical structure with a tip as a reference point for accurate targeting of the focus. By using the same 3D-printing resin as that of the lens, acoustic attenuation was minimized through the homogeneous medium. The collimator was integrated with the acoustic lens in the 3D model, and the lens was designed based on the time-reversal recording process with the geometric and material properties of the collimator. Finally, the collimated acoustic lens was fabricated by 3D-printing (Fig. 5a). In our scheme, alignment errors were minimized by omitting an alignment step of the collimator with the lens, and propagation loss was similarly minimized by reducing the number of interfaces in the transmission path. The collimated acoustic lens and the CMUT were combined using silicone elastomer (Sylgard 184, Dow, USA) and packaged with a lightweight 0.2-mm-thick printed circuit board (PCB). To minimize the effect of tethering on mouse behavior during stimulation, flexible copper wires were soldered to the PCB for CMUT actuation. The final weight of the integrated device was only 0.75 g, which was tenfold lighter than a commercial PUT with the same aperture diameter of 8 mm (Fig. 5b).

### In vivo characterization of the package

To evaluate the effects of the attached package on mouse behavioral studies, the freely moving behaviors of mice with the package affixed to the head were analyzed. The packaged device was affixed to the mouse skull through surgical adhesion for targeted brain stimulation (Fig. 5c). The collimated acoustic lens was aligned to the target region using stereotaxic coordinates and fixed onto the skull using biocompatible adhesives to achieve robust packaging for behavioral studies. Prior to the experiments, the mice were given 7–10 days for recovery postsurgery. We compared three experimental groups: (1) without device and surgery (Normal), (2) without device but with sham surgery (Sham), and (3) with device and surgery (Device) (Fig. 5d). The activity of the mice was recorded using a smart home camera in an open field (30 × 30 cm^2^), and the trajectory of mouse motion was analyzed using DeepLabCut for quantification of the activity level (Fig. 5e)^46^. There was no statistically significant change in the movement of mice in terms of speed and cumulative travel distance due to the minimal weight of the package (Figs. 5f, g, S10, S11; ns, one-way ANOVA with Tukey’s post hoc test, *p* > 0.05, *n* = 9).

A mouse skull was harvested ex vivo and packaged with the device according to the same coordinate-based alignment protocol as the in vivo experiment. Then, the acoustic pressure profile was directly measured by scanning the needle hydrophone within the half-skull cavity (Fig. S12). The profile agreed well with the simulation results. The peak pressure amplitude was 44.6 kPa with a DC bias of 42 V and peak-to-peak AC swing of 5 V, which was sufficient for neuromodulation^55^. However, considering that this peak pressure value was obtained from focusing, the acoustic pressure level was on the same order as the planar wave from the bare CMUT (Fig. 4g, h). One major reason for this observation is the large difference in acoustic impedance at the material interface in the lens (Z = 3.03 MRayl for the 3D-printed resin and Z = 1.1 MRayl for the PDMS, resulting in a reflection coefficient of 0.22). This high reflection coefficient not only reduces the acoustic pressure delivered at the focal point but also interferes with the phase map for focal reconstruction by the reflected acoustic field. Even so, further investigation of optimized material pairs for lens construction is left to future work. While we demonstrated our system at an ultrasound frequency of 2 MHz, it can be readily expanded to different ultrasound frequencies devising a new design of CMUT with the same footprint. In future work, the acoustic property distribution of mouse skulls must be measured and established for different ages of mice, to facilitate precise in vivo transcranial focused ultrasound stimulation.

### Limitations and future work

Our work leaves room for several future improvement. First, the acoustic pressure of the device was not high enough for versatile applications that require higher pressure (such as blood‒brain-barrier (BBB) opening)^56^. Instead of PDMS and the 3D-printed resin, other pairs of acoustic lens materials that are compatible with the CMUT and offer a smaller difference in acoustic impedance should be used to achieve higher pressure. Second, the focal size of the 2 MHz ultrasound beam in our work, which was 3 mm in the axial dimension, was not small enough to target small subregions of the brain. For applications targeting complex brain circuits and related subregions to treat brain disorders such as Parkinson’s disease or depression, we must improve the focal resolution^57–59^. Increasing the aperture size from 8 mm can improve the axial resolution of the focus but will also increase its size. Thus, a new design for a CMUT with a resonance frequency of 5 MHz or higher is needed. Third, the current system tethers mice to power the CMUT. There are certain behavioral experiments that are not possible with tethering, such as running wheel experiments. For such systems, the integration of a wireless driving circuit for the CMUT might be necessary. Fourth, because our system is fixed on the skull, we cannot steer the lens after surgery. Nevertheless, the same design of acoustic lenses can be utilized for different target regions during surgery. Finally, although ultrasound neuromodulation is ideally noninvasive, a scalp incision was needed in this study. Because targeting a specific region in the brain relies on bregma on the skull as a reference point for the guiding coordinate, most works on ultrasound neuromodulation fix the stimulation system on the skull after scalp removal. In the future, we must find a method to target a subregion without referencing the skull. Because CMUTs are more compatible with MR imaging than PUTs^17^, we believe that we will be able to develop a targeting system using fMRI^60,61^.

## Conclusions

In this work, we demonstrated a miniaturized transcranial ultrasound focusing system for targeted neuromodulation in a freely moving mouse. To accomplish miniaturization, we integrated the 3D-printed acoustic lens with a lightweight micromachined transducer as a single acoustic source. The acoustic lens was designed using the principle of a phase conjugation mirror with numerical simulation of the time-reversal recording through the skull, and the system was validated using a skull phantom and a commercial piezoelectric ultrasound transducer at 5 MHz. The CMUT was analytically and numerically designed for neuromodulation applications in terms of the frequency and acoustic pressure of the transmitted ultrasound beam. Direct wafer bonding and silicon micromachining technology enabled wafer-scale fabrication of the CMUT. By manipulating the acoustic wavefront from the planar acoustic source of the CMUT using the acoustic lens, a miniaturized novel device for transcranial ultrasound focusing was demonstrated. With the minimized weight of the device being 0.75 g, we observed no significant changes in mouse behavior when our packaged device was attached to the mouse skulls. Using the proposed system, investigation of the efficacy and mechanisms of ultrasound neuromodulation therapies for brain diseases is possible by accurately targeting neural circuits.

## Supplementary information

Supplementary material

## References

[1] Baek H, Pahk KJ, Kim H (2017). A review of low-intensity focused ultrasound for neuromodulation. Biomed. Eng. Lett..

[2] Kamimura HAS, Conti A, Toschi N, Konofagou EE (2020). Ultrasound neuromodulation: Mechanisms and the potential of multimodal stimulation for neuronal function assessment. Front. Phys..

[3] Blackmore J, Shrivastava S, Sallet J, Butler CR, Cleveland RO (2019). Ultrasound neuromodulation: A review of results, mechanisms and safety. Ultrasound Med. Biol..

[4] Fouragnan EF (2019). The macaque anterior cingulate cortex translates counterfactual choice value into actual behavioral change. Nat. Neurosci..

[5] Tufail Y (2010). Transcranial pulsed ultrasound stimulates intact brain circuits. Neuron.

[6] Hakimova H (2015). Ultrasound stimulation inhibits recurrent seizures and improves behavioral outcome in an experimental model of mesial temporal lobe epilepsy. Epilepsy Behav..

[7] Min BK (2011). Focused ultrasound-mediated suppression of chemically-induced acute epileptic eeg activity. BMC Neurosci..

[8] Yang FY, Lu WW, Lin WT, Chang CW, Huang SL (2015). Enhancement of neurotrophic factors in astrocyte for neuroprotective effects in brain disorders using low-intensity pulsed ultrasound stimulation. Brain Stimul..

[9] Kim E (2020). Wearable transcranial ultrasound system for remote stimulation of freely moving animal. IEEE Trans. Biomed. Eng..

[10] Lee W (2016). Transcranial focused ultrasound stimulation of human primary visual cortex. Sci. Rep..

[11] Folloni D (2019). Manipulation of subcortical and deep cortical activity in the primate brain using transcranial focused ultrasound stimulation. Neuron.

[12] Wang J (2019). Transcranial focused ultrasound stimulation regulates brain plasticity in mice after middle cerebral artery occlusion. J. Cereb. Blood Flow. Metab..

[13] Kim S (2021). Transcranial focused ultrasound stimulation with high spatial resolution. Brain Stimul..

[14] Marino A (2015). Piezoelectric nanoparticle-assisted wireless neuronal stimulation. Acs Nano.

[15] Zhao D (2020). Electromagnetized-nanoparticle-modulated neural plasticity and recovery of degenerative dopaminergic neurons in the mid-brain. Adv. Mater..

[16] Wu X (2019). Sono-optogenetics facilitated by a circulation- delivered rechargeable light source for minimally invasive optogenetics. Proc. Natl Acad. Sci. USA.

[17] Kim H (2019). Miniature ultrasound ring array transducers for transcranial ultrasound neuromodulation of freely-moving small animals. Brain Stimul..

[18] Jo Y (2022). General-purpose ultrasound neuromodulation system for chronic, closed-loop preclinical studies in freely behaving rodents. Adv. Sci..

[19] Li GF (2019). Noninvasive ultrasonic neuromodulation in freely moving mice. IEEE Trans. Biomed. Eng..

[20] Lee W (2018). Transcranial focused ultrasound stimulation of motor cortical areas in freely-moving awake rats. BMC Neurosci..

[21] Bystritsky A (2011). A review of low-intensity focused ultrasound pulsation. Brain Stimul..

[22] Yu K, Niu XD, Krook-Magnuson E, He B (2021). Intrinsic functional neuron-type selectivity of transcranial focused ultrasound neuromodulation. Nat. Commun..

[23] Li GF (2016). Improved anatomical specificity of non-invasive neuro-stimulation by high frequency (5 mhz) ultrasound. Sci. Rep..

[24] Aubry JF, Tanter M, Pernot M, Thomas JL, Fink M (2003). Experimental demonstration of noninvasive transskull adaptive focusing based on prior computed tomography scans. J. Acoustical Soc. Am..

[25] Couture O, Aubry JF, Tanter M, Fink M (2009). Time-reversal focusing of therapeutic ultrasound on targeted microbubbles. Appl. Phys. Lett..

[26] De Angelis A (2021). Computational optimization of transcranial focused ultrasound stimulation: Toward noninvasive, selective stimulation of deep brain structures. Appl. Phys. Lett..

[27] Rahimi S, Jones RM, Hynynen K (2021). A high-frequency phased array system for transcranial ultrasound delivery in small animals. Ieee Trans. Ultrason. Ferroelectr. Frequency Control.

[28] Adams C (2021). Implementation of a skull-conformal phased array for transcranial focused ultrasound therapy. IEEE Trans. Biomed. Eng..

[29] Chen SG (2020). Transcranial focused ultrasound pulsation suppresses pentylenetetrazol induced epilepsy in vivo. Brain Stimul..

[30] Melde K, Mark AG, Qiu T, Fischer P (2016). Holograms for acoustics. Nature.

[31] Maimbourg G, Houdouin A, Deffieux T, Tanter M, Aubry JF (2018). 3d-printed adaptive acoustic lens as a disruptive technology for transcranial ultrasound therapy using single-element transducers. Phys. Med. Biol..

[32] He JR (2022). Multitarget transcranial ultrasound therapy in small animals based on phase-only acoustic holographic lens. Ieee Trans. Ultrason. Ferroelectr. Freq Control.

[33] Seok C, Yamaner F. Y., Sahin M., Oralkan Ö. A sub-millimeter lateral resolution ultrasonic beamforming system for brain stimulation in behaving animals. 2019 41st Annual International Conference of the IEEE Engineering in Medicine and Biology Society (EMBC). (2019) 10.1109/EMBC.2019.8857627 31947322

[34] Jones RM, Caskey CF, Dayton PA, Oralkan Ö, Pinton GF (2021). Transcranial neuromodulation array with imaging aperture for simultaneous multifocus stimulation in nonhuman primates. IEEE Trans. Ultrason. Ferroelectr. Freq COntrol..

[35] Khuri-Yakub BT, Oralkan O (2011). Capacitive micromachined ultrasonic transducers for medical imaging and therapy. J. Micromech. Microeng..

[36] Treeby BE, Cox BT (2010). K-wave: Matlab toolbox for the simulation and reconstruction of photoacoustic wave fields. J. Biomed. Opt..

[37] Treeby BE, Jaros J, Rendell AP, Cox BT (2012). Modeling nonlinear ultrasound propagation in heterogeneous media with power law absorption using a k-space pseudospectral method. J. Acoust. Soc. Am..

[38] Treeby B. E., Jaros J., Rohrbac D., Cox B. T. Modelling elastic wave propagation using the k-wave matlab toolbox. 2014 Ieee International Ultrasonics Symposium (Ius). 146–149. 10.1109/Ultsym.2014.0037 (2014).

[39] Treeby BE, Budisky J, Wise ES, Jaros J, Cox BT (2018). Rapid calculation of acoustic fields from arbitrary continuous-wave sources. J. Acoust. Soc. Am..

[40] Bojarski NN (1982). The k-space formulation of the scattering problem in the time domain. J. Acoust. Soc. Am..

[41] Bojarski NN (1985). The k-space formulation of the scattering problem in the time domain—an improved single propagator formulation. J. Acoust. Soc. Am..

[42] Mast TD (2001). A kappa-space method for large-scale models of wave propagation in tissue. Ieee Trans. Ultrason. Ferroelectr. Freq. Control.

[43] Tabei M, Mast TD, Waag RC (2002). A k-space method for coupled first-order acoustic propagation equations. J. Acoust. Soc. Am..

[44] Estrada H, Rebling J, Turner J, Razansky D (2016). Broadband acoustic properties of a murine skull. Phys. Med. Biol..

[45] Park KK, Lee H, Kupnik M (2011). B. T. Khuri-Yakub. Transcranial focused ultrasound stimulation regulates brain plasticity in mice after middle cerebral artery occlusion. J. Microelectromechan. Syst..

[46] Mathis A (2018). Deeplabcut: Markerless pose estimation of user-defined body parts with deep learning. Nat. Neurosci..

[47] Ngo TD, Kashani A, Imbalzano G, Nguyen KTQ, Hui D (2018). Additive manufacturing (3d printing): A review of materials, methods, applications and challenges. Compos. Part B-Eng..

[48] Kurimoto M., Manabe Y., Mitsumoto S., Suzuoki Y. Layer interface effects on dielectric breakdown strength of 3d printed rubber insulator using stereolithography. Additive Manufacturing. **46**. 10.1016/j.addma.2021.102069 (2021).

[49] de Haas R (2012). Wireless implantable micro-stimulation device for high frequency bilateral deep brain stimulation in freely moving mice. J. Neurosci. Methods.

[50] Maimbourg G, Houdouin A, Deffieux T, Tanter M, Aubry JF (2020). Steering capabilities of an acoustic lens for transcranial therapy: Numerical and experimental studies. IEEE Trans. Biomed. Eng..

[51] Gross D, Coutier C, Legros M, Bouakaz A, Certon D (2015). A cmut probe for ultrasound-guided focused ultrasound targeted therapy. Ieee Trans. Ultrason. Ferroelectr. Freq. Control.

[52] Park K. K. et al. Optimum design of circular cmut membranes for high quality factor in air. 2008 Ieee Ultrasonics Symposium, Vols 1-4 and Appendix. 504–507. 10.1109/Ultsym.2008.0123 (2008).

[53] Anbalagan SA, Uma G, Umapathy M (2006). Modeling and simulation of capacitive micromachined ultrasonic transducer (cmut). Int. Mems Conf. 2006.

[54] Lee H. J. et al. A low-noise oscillator based on a multi-membrane cmut for high sensitivity resonant chemical sensors. Ieee 22nd International Conference on Micro Electro Mechanical Systems (Mems 2009). 761–764. 10.1109/Memsys.2009.4805494 (2009).

[55] Pasquinelli C, Hanson LG, Siebner HR, Lee HJ, Thielscher A (2019). Safety of transcranial focused ultrasound stimulation: A systematic review of the state of knowledge from both human and animal studies. Brain Stimul..

[56] Konofagou EE (2012). Ultrasound-induced blood-brain barrier opening. Curr. Pharm. Biotechnol..

[57] Kravitz AV (2010). Regulation of parkinsonian motor behaviours by optogenetic control of basal ganglia circuitry. Nature.

[58] Tye KM, Deisseroth K (2012). Optogenetic investigation of neural circuits underlying brain disease in animal models. Nat. Rev. Neurosci..

[59] Muir J, Lopez J, Bagot RC (2019). Wiring the depressed brain: Optogenetic and chemogenetic circuit interrogation in animal models of depression. Neuropsychopharmacol.

[60] Ai L, Bansal P, Mueller JK, Legon W (2018). Effects of transcranial focused ultrasound on human primary motor cortex using 7t fmri: A pilot study. BMC Neurosci..

[61] Yang PF (2021). Bidirectional and state-dependent modulation of brain activity by transcranial focused ultrasound in non-human primates. Brain Stimul..

