# Supplementary material for: Multifocal skull-compensated transcranial focused ultrasound system for neuromodulation applications based on acoustic holography
Source: Microsyst Nanoeng. 2023 Apr 10;9:45. doi: 10.1038/s41378-023-00513-3 (PMC10085992; doi:10.1038/s41378-023-00513-3)
Supplement: Supplementary file 1 — Supplementary material [file 41378_2023_513_MOESM1_ESM.docx]

Supplementary Information

Multifocal Skull-Compensated Transcranial Focused Ultrasound System for Neuromodulation Applications based on Acoustic Holography

Geon Kook, Yehhyun Jo, Chaerin Oh, Xiaojia Liang, Jaewon Kim, Sang-Mok Lee, Subeen Kim, Jung-Woo Choi, and Hyunjoo Jenny Lee*


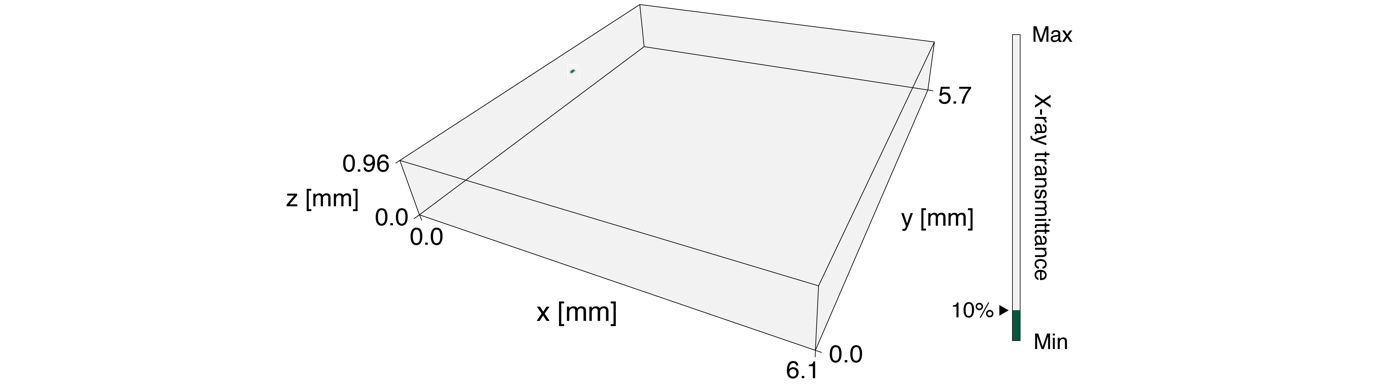


Fig. S1 Void visualization of the acoustic lens using the X-ray microscope in a volume of 6.1 × 5.7 × 0.96 mm^3^. Green dots indicate the void, which was 0.001% of the total volume. Focal size of the X-ray was 50 µm, and the threshold for the void was the lower 10 % transmittance in the volume


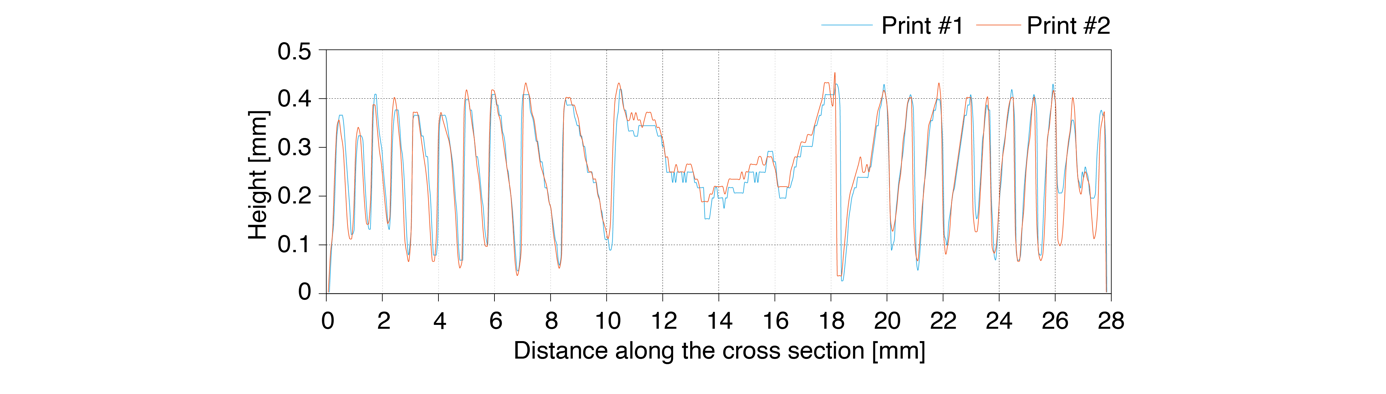


Fig. S2 Surface profiles along a fixed line in two different samples printed using the same 3D model


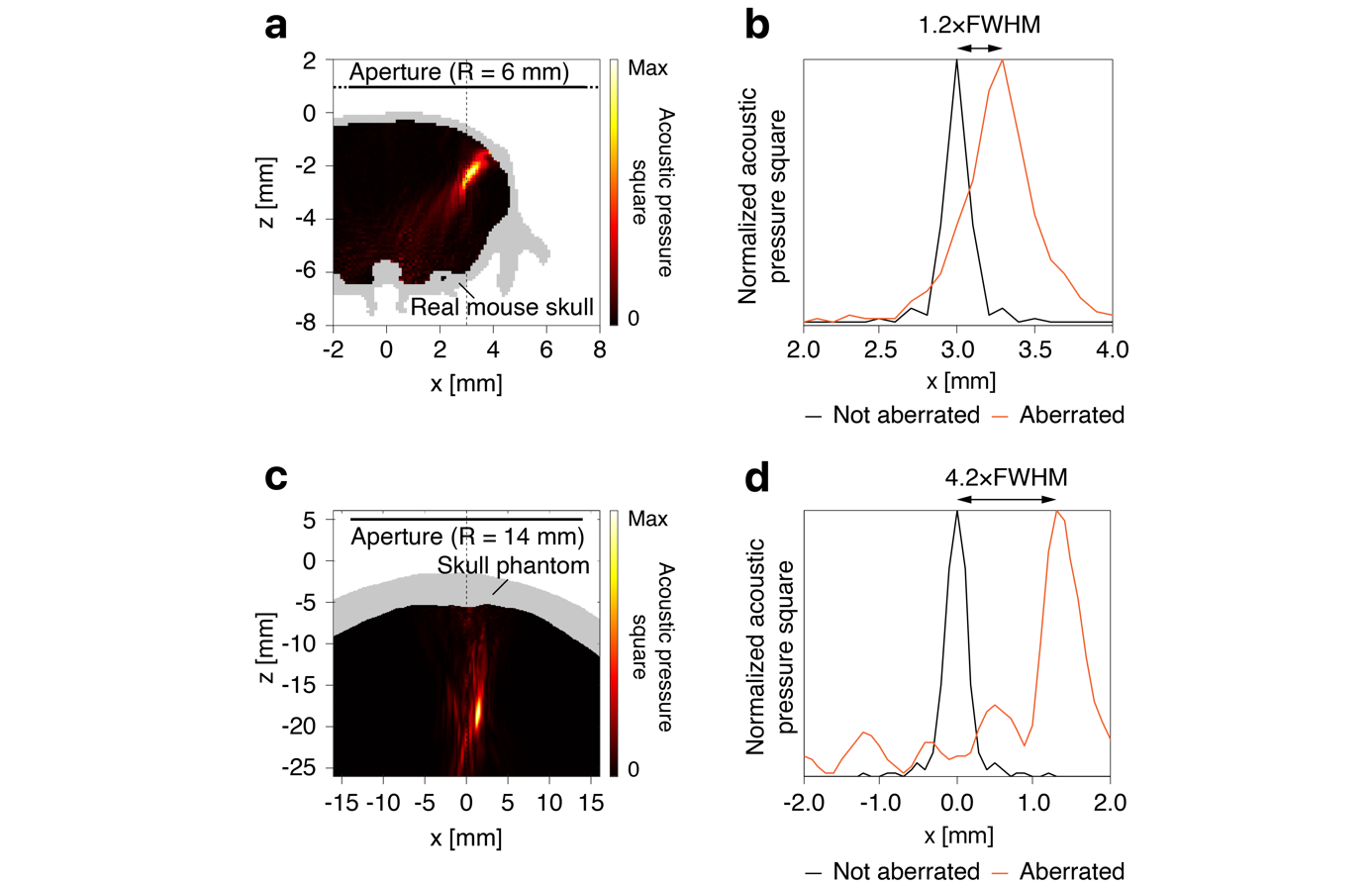


Fig. S3 a Simulated focal aberration by a real mouse skull using a free-space-focusing source of aperture diameter of 12 mm. b Comparison of the profile in a with the ideal focal profile (not aberrated). c Simulated focal aberration by the skull phantom using a free-space-focusing source of aperture diameter of 28 mm. d Comparison of the profile in c with the ideal focal profile (not aberrated)


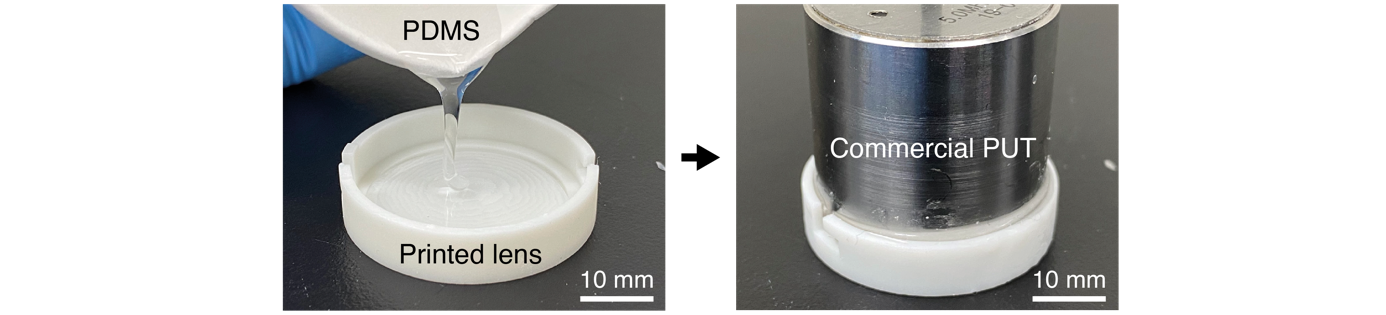


Fig. S4 Packaging of the printed acoustic lens with the commercial piezoelectric ultrasound transducer (PUT) for the evaluation


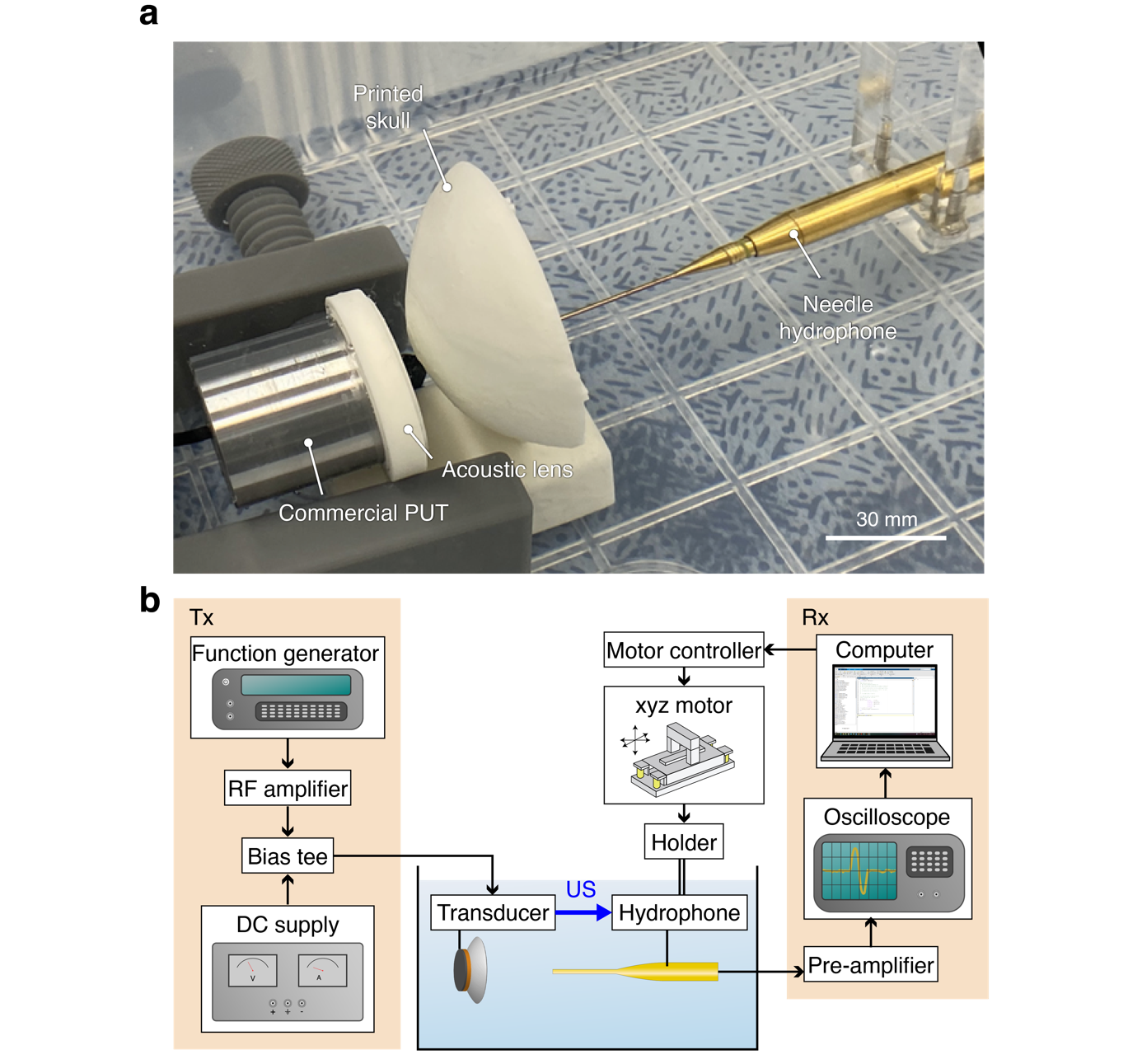


Fig. S5 a A photograph of the experimental setup to evaluate the fabricated acoustic lens using the skull phantom and the needle hydrophone. b Block diagram of the measurement system


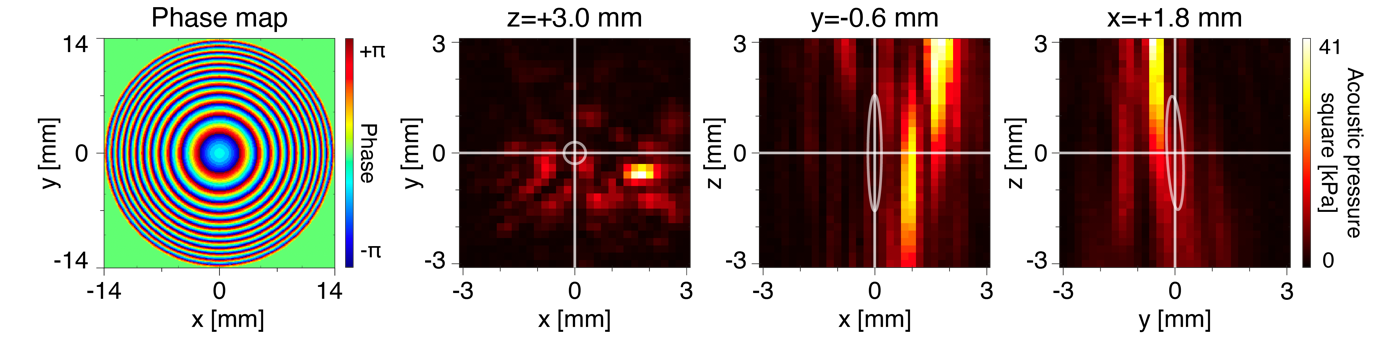


Fig. S6 Phase design of the Fresnel acoustic lens and the skull phantom-aberrated acoustic pressure square profile from the fabricated lens


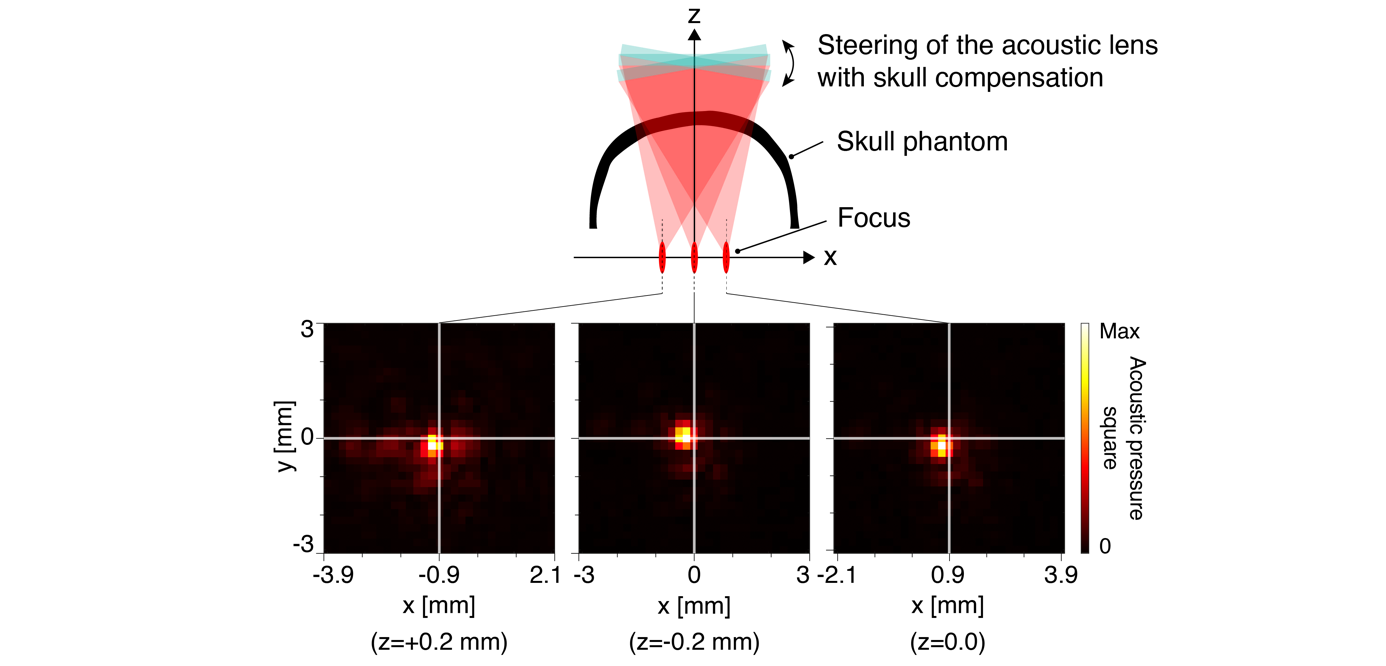


Fig. S7 Acoustic pressure square profile from the same acoustic lens at different steering angles targeting ± 0.9 mm away from the center along the x-axis


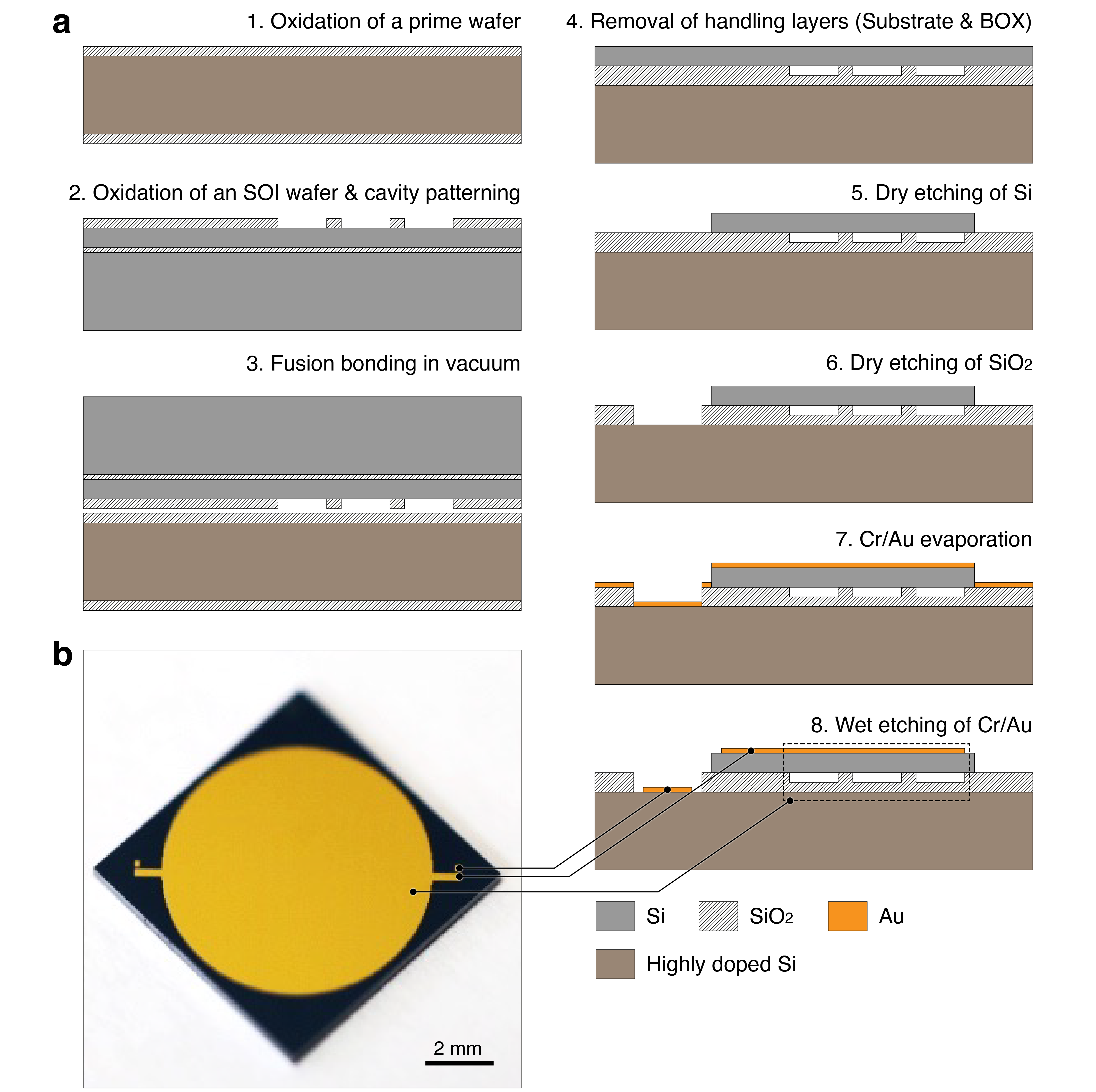


Fig. S8 a Fabrication process of the CMUT based on the wafer direct bonding method. b A photograph of the final device


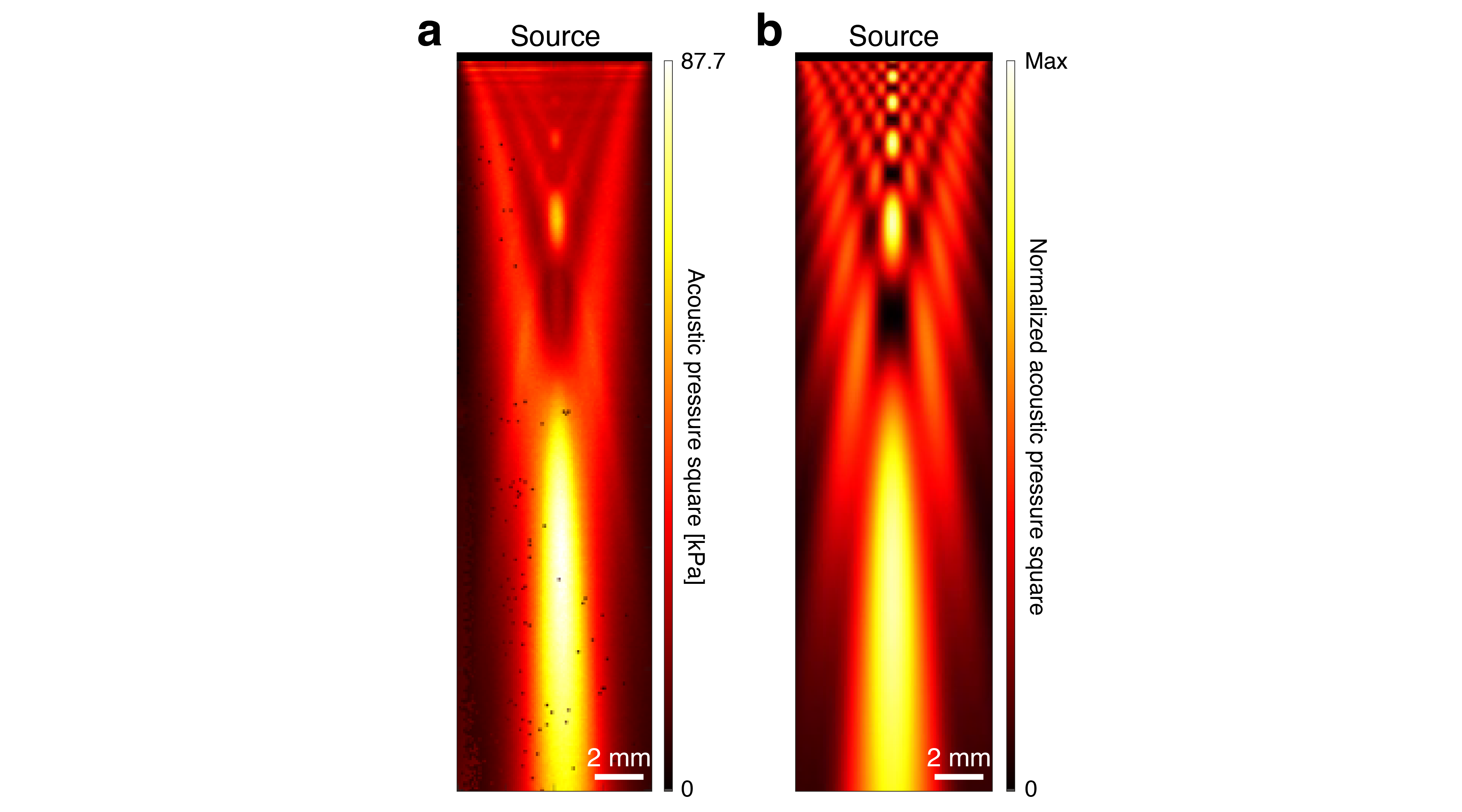


Fig. S9 a Free-space beam profile of the fabricated CMUT in oil biased with DC voltage of 42 V and AC voltages of 10 Vpp at 2 MHz. b A simulated beam profile from an ideal plane source with the same aperture diameter


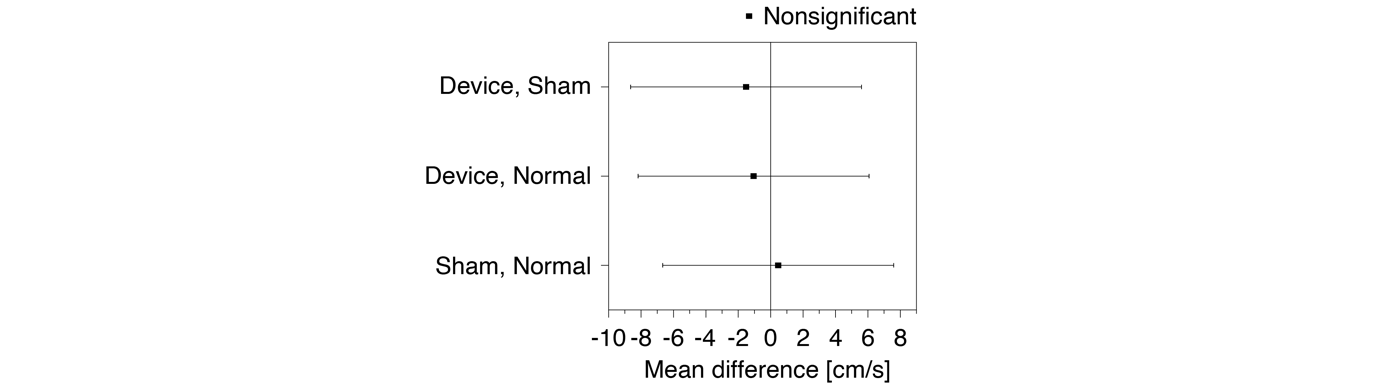


Fig. S10 Multiple comparison plots using the experimental groups as the factor for the average speed of mice using one-way ANOVA with Tukey’s post-hoc test. Three experimental groups were (1) without device and surgery (Normal), (2) without device but with sham surgery (Sham), and (3) with device and surgery (Device)


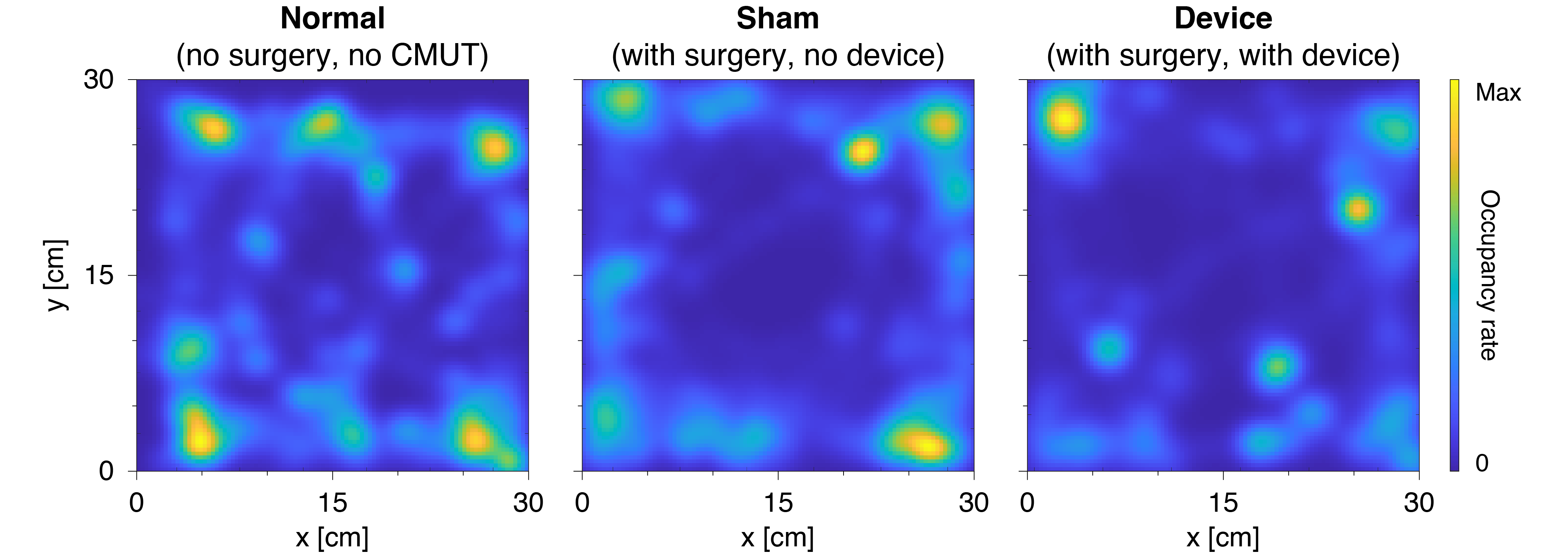


Fig. S11 Representative images of the occupancy rate of mice in the open field for three experimental groups

**
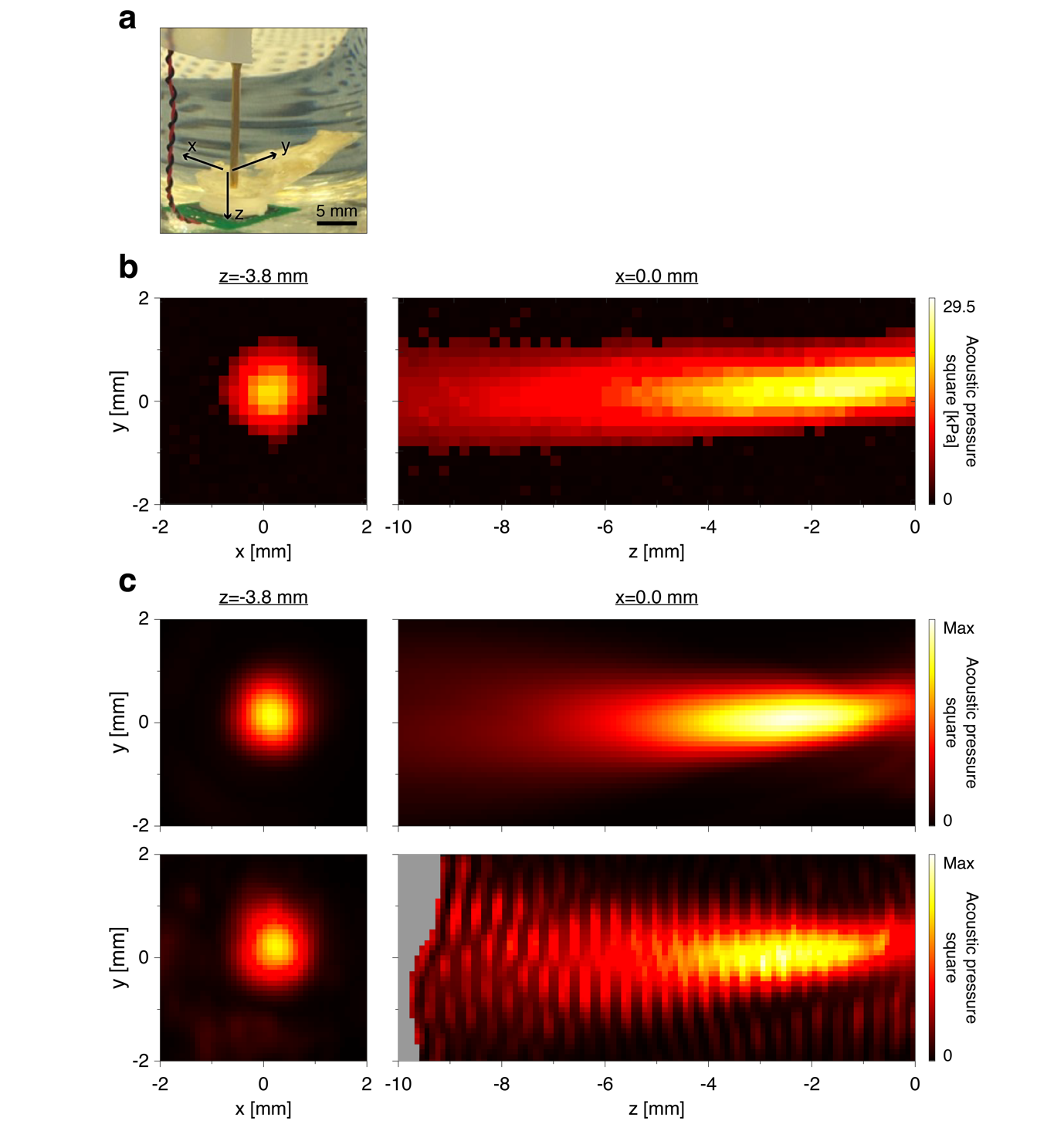
**

Fig. S12 a A photograph of the measurement system of the acoustic profile through the skull where a real mouse skull was used. b Acoustic pressure square profile from the CMUT-acoustic lens package through the mouse skull. c Ideal simulated acoustic pressure square profile of b without (up) and with (down) the lower part of the skull. Grey area represents the lower part of the skull in the simulation, which was removed during the real measurement
